# Density-equalizing mapping and scientometric benchmarking of European allergy research

**DOI:** 10.1186/1745-6673-5-2

**Published:** 2010-02-16

**Authors:** Cristian Scutaru, David Quarcoo, Mohannad Sakr, Awfa Shami, Khaled Al-Mutawakel, Karin Vitzthum, Tanja C Fischer, Torsten Zuberbier, Beatrix Groneberg-Kloft

**Affiliations:** 1 Institute of Occupational Medicine, Charité-Universitätsmedizin Berlin, Free University Berlin and Humboldt-University Berlin, Berlin, Germany; 2 Department of Respiratory Medicine, Hanover Medical School, Hanover, Germany; 3 Allergy-Centre-Charité, Department of Dermatology and Allergy, Charité-Universitätsmedizin Berlin, Free University Berlin and Humboldt-University Berlin, Berlin, Germany; 4 Otto-Heubner-Centre, Charité-Universitätsmedizin Berlin, Free University Berlin and Humboldt-University Berlin, Berlin, Germany

## Abstract

Due to the great socioeconomic burden of allergic diseases, research in this field which is important for environmental medicine is currently increasing. Therefore the European Union has initiated the Global Allergy and Asthma European network (GA2LEN). However, despite increasing research in the past years detailed scientometric analyses have not been conducted so far. This study is the first scientometric analysis in a field of growing interest. It analyses scientific contributions in European allergy research between 2001 and 2007. Three different meetings of the European Academy of Allergy and Clinical Immunology were analysed for contributions and an increase in both the amount of research and networks was found.

## Introduction

Allergic diseases are complex inflammatory conditions with an increasing prevalence and incidence [1]. They play a major role in environmental and occupational medicine and encompass i.e. allergic bronchial asthma [2], allergic rhinitis [3], atopic dermatitis [4], food allergy [5] and allergic eye diseases [6]. The direct medical costs evolved from allergic diseases are increasing over the past decades. I.e. bronchial asthma constitutes about an estimated 1-3% of the health fund of the U.S and the economic burden amounts to roughly 12 billion dollar [7-9]. Also other allergic diseases exert a major toll on the heath care systems [10]. Despite the large amount of clinical and experimental studies already conducted on allergic diseases, further insights into the molecular basics are required to develop new therapeutic strategies.

Therefore, the European Union (EU) started a network of excellence, bringing together epidemiological and clinical researchers who investigate allergy and asthma across the life stages. Launched as a Network of Excellence of 25 leading European teams as well as the European Academy of Allergology and Clinical Immunology (EAACI) and the European Federation of Allergy and Airways Diseases Patients Associations (EFA) on February 2004, a total budget of EUR 14.4 million for a five-year period has been allocated from the EU's Sixth Research Framework Programme for GA2LEN activities [11]. GA^2^LEN's research examines new ways of understanding, preventing and managing allergies and asthma. Research activities focus on epidemiology; early life events in the development of sensitisation; the translation of allergic sensitisation into allergic disease; the persistence in aggravation of allergic diseases and asthma. The collaboration project reflects recognition of growing concern among European citizens about rising rates of allergy and asthma [12]. In this respect the scientific community depends on annual meetings to propagate the novel insights. The European Academy of Allergy and Clinical Immunology (EAACI) has already 26 annual congresses. However, there is no in-depth scientometric analysis of allergy research available so far. Therefore the present study was carried out to evaluate European allergy research using these congresses in the light of a growing interest in allergy research. Bibliometric approaches in combination with density-equalizing mapping were used for this purpose.

We hypothesized that the growing interest in allergy research is reflected by the output of abstracts at the largest European allergy meetings. Furthermore we were interested in investigating whether the creation of research networks such as the GA2LEN might influence scientific cooperation among EU countries.

## Methods

### Data source

Data was retrieved from the over 1500 pages of the proceedings books of the three different annual meetings in Berlin, Vienna and Gotenborg [13].

### Data acquisition

Data from the abstract books was entered into Excel spreadsheets following the subsequent rules: 1) The country from which the author of the article comes is inserted into the database program (Excel) 2) If an article is a result from a multinational cooperation (authors from different countries) each land is inserted in to the database program.

### Data analysis

The following parameters were screened: 1) Number of abstracts originating from a specific country. 2) National interests for the field of allergy over the years (number of abstracts over the years) 3) Global analysis of the participations using density equalizing maps 4) Development of international cooperations in the allergy research.

A table was subsequently generated with all the participant countries and the number of papers coming from that country using Visual Basic for Applications (VBA).

### Density-equalizing mapping

Density-equalizing mapping was used as described previously [14-16]. In brief, territories were re-sized according to a particular variable, i.e. the number of published items at the three congresses. For the re-sizing procedure the area of each country was scaled in proportion to its total number of published items. The specific calculations are based on Gastner and Newman's algorithm [17].

### Analysis of bilateral and multinational cooperations

A bilateral cooperation between 2 countries was defined when at least one author originates from one country and at least one other author from a second country. A matrix with all participant countries was computed with special software and filled with the appropriate values for the cooperation for each pair of countries. A software program was developed to interpret the matrix and transform the figures into vectors. The thickness of a vector quantifies the cooperation between the two countries. A threshold was also programmed in order to filter low numbers of bilateral cooperations (i.e. less then 5).

## Results

### Total number of published items

**Figure 1:**
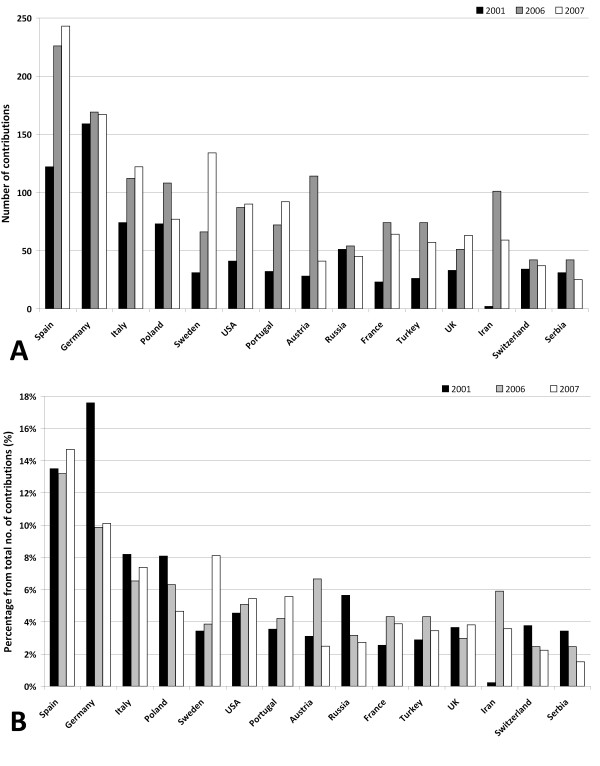
**Published items at the annual meetings of the European Academy of Allergy and Clinical Immunology**. A total number of contributions at the Berlin (2001), Vienna (2006) and Gotenborg (2007) meetings. B percentage of contribution for each meetings.

**Figure 2:**
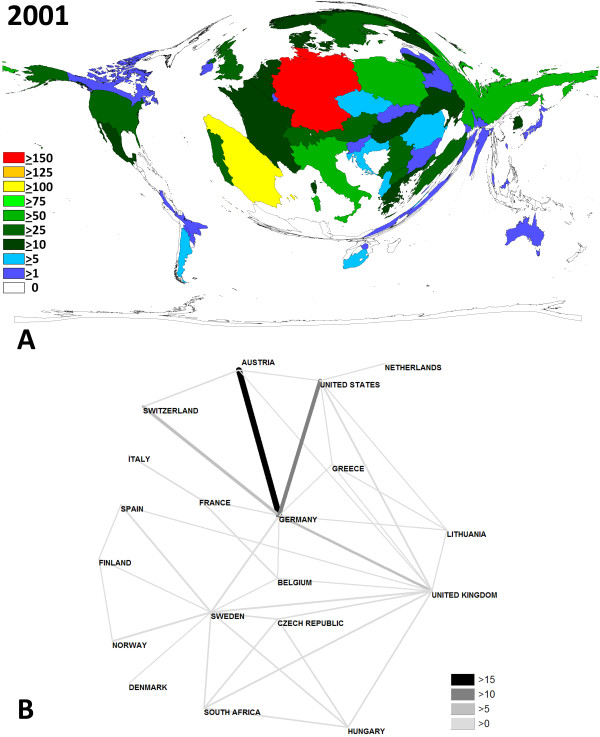
**Contribution and network analysis for the 2001 meeting**. A Density-equalizing map illustrating the number of contributions for each country. The area of each country was scaled in proportion to its total number of publications regarding the contributions. Colors encode the number of contributions per country. B Chart visualizing the networking. Greyscale and size of bars encode the number of bilateral cooperations.

The number of published items was used as an index of quantity of research productivity and large differences were found: At the 2001 annual meeting 904 items were published by 58 countries (Fig. 1). At the 2006 meeting 1713 items were published by 75 countries and at the 2007 annual meeting 1653 items were published by 74 countries (fig. 2). The most productive countries were in 2001 Germany (159 items) followed by Spain (122 items), Italy (74 items), Poland (73 items) and the Russian Federation (51 items) while in 2006, Spain was the most productive country with 226 published items followed by Germany (169 items), Austria (114 items), Italy (112) and Poland (112). At the last congress in Sweden, Spain was again the most productive country (243 items) followed by Germany (167 items), Sweden (134 items), Portugal (122 items) and Italy (92 items).

Assessment of trends for single countries between the three congresses illustrates different trends: A general increase in numbers of published items can be seen. Countries including Poland, Austria, Iran, France and Turkey had a prominent contribution at the 2006 meeting and decreased numbers of published items in 2007 (Fig. 1).

### Density equalizing mapping

**Figure 3:**
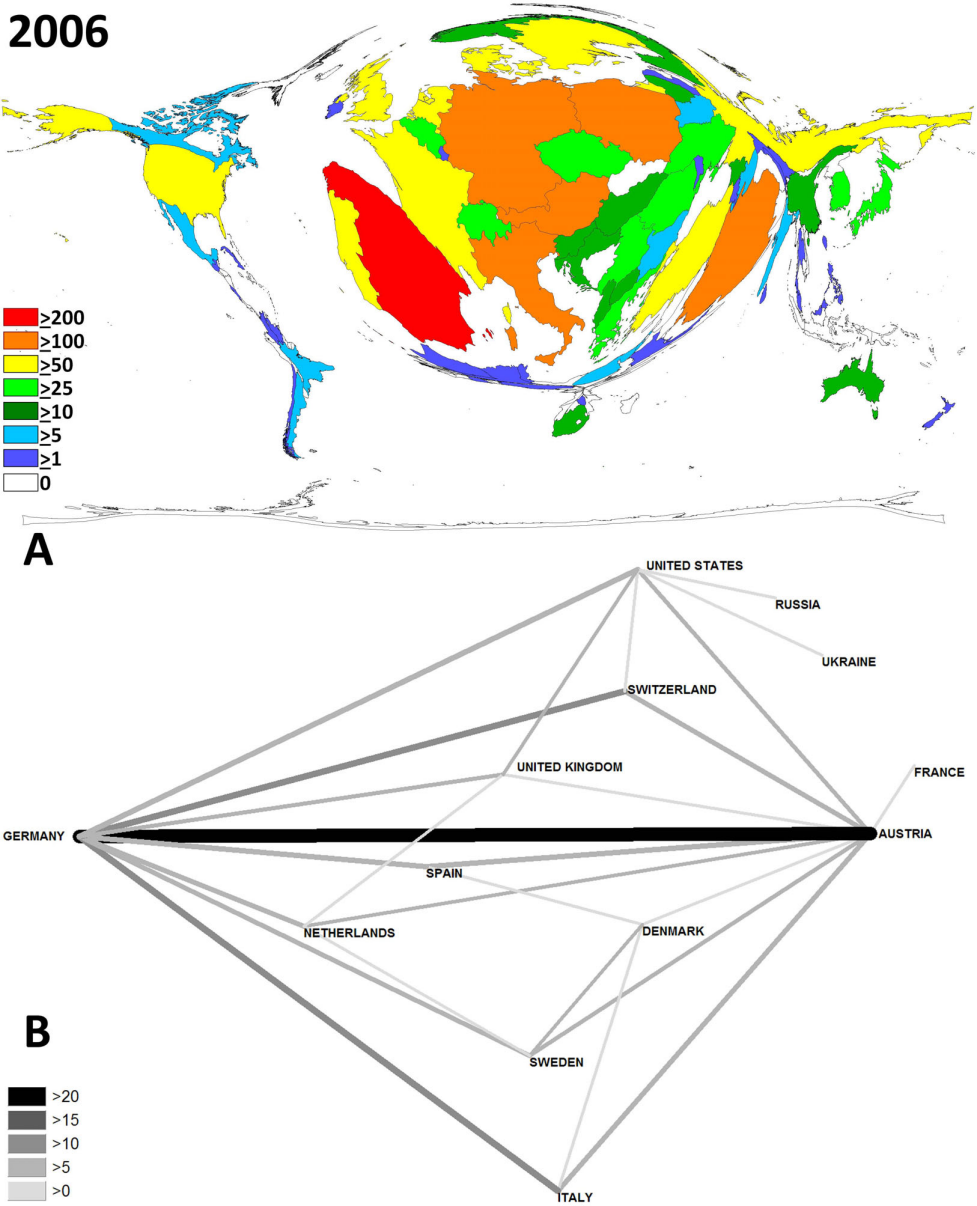
**Contribution and network analysis for the 2006 meeting**. A Density-equalizing map illustrating the number of contributions for each country. The area of each country was scaled in proportion to its total number of publications regarding the contributions. Colors encode the number of contributions per country. B Chart visualizing the networking. Greyscale and size of bars encode the number of bilateral cooperations.

**Figure 4:**
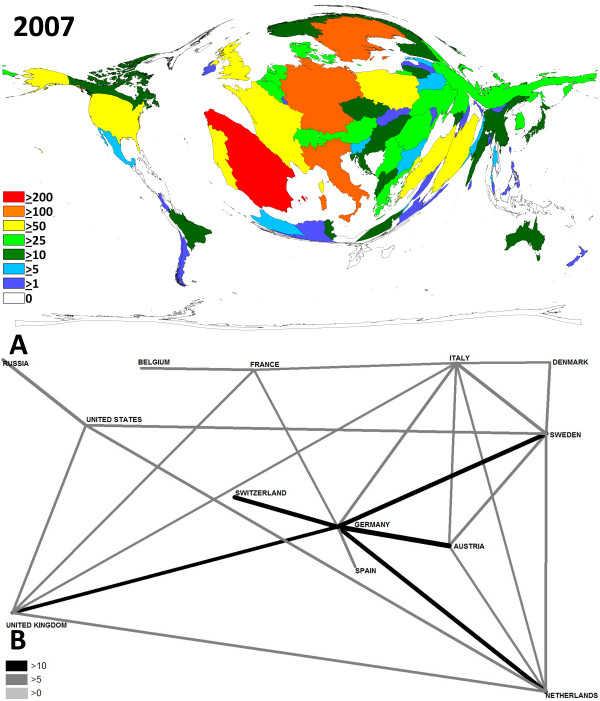
**Contribution and network analysis for the 2007 meeting**. A Density-equalizing map illustrating the number of contributions for each country. The area of each country was scaled in proportion to its total number of publications regarding the contributions. Colors encode the number of contributions per country. B Chart visualizing the networking. Greyscale and size of bars encode the number of bilateral cooperations.

Density-equalizing mapping was used according to a recently published method to illustrate focuses of research by territorial resizing. It was found that the hosting countries dominated the meetings in terms of numbers of presented studies: In every meeting in Germany (Fig. 2), Austria (Fig. 3) and Sweden (Fig. 4), they were listed within the top 3 most productive countries. Apart from this host-dependant trend, Spain was also very active.

### Networks

The biggest change lies in the cooperation between different countries. This can probably be explained due the faster and cheaper means of communications and the increasing founding of European networks. In specific, at the 2001 meeting in Munich, networks were dominant between Germany and the US and Germany and Austria. However, the total numbers of bilateral cooperations (> 10 bilateral) were little (Fig. 2). An increase of bilateral cooperation (number > 10) was found for the 2006 meeting in Sweden. Here German-Austrian networks dominated (Fig. 3). At the 2007 meeting, German scientists again had the highest number of bilateral cooperations (Fig. 4).

## Discussion

The present study is the first analysis to assess allergy research progress using scientometric methods in combination with density-equalizing mapping procedures. An increasing number of networks was found when 2001 scientific activities were compared to 2006 and 2007. This needs to be interpreted in the context of allergy research and funding: Allergic diseases are estimated to cost Europe about 25 billion Euros annually, with most of the costs due to reduced productivity in school or at work and there is in increasing burden of allergic diseases present in Europe. Therefore, the European Union started the GA2LEN network with the intention to establish a world-wide competitive network of European centres of excellence in order to enhance the quality and relevance of research in the area of allergy and to address allergy and asthma in their totality. The research network focuses its research programme on developing new ways of preventing and managing allergies and asthma. In the long term, the research network aims also to decrease the socioeconomic burden of allergy and asthma in Europe. In close association to these aims are the meetings of the European Academy of Allergy and Clinical Immunology. They provide substantial progress in knowledge along with offering the opportunity for scientific networking and exchanging ideas. Due to the fact that there has been such an inflow of information surrounding allergy, it was decided in 1999 that there would be a four-day Congress every year. Generally, there is a constant increase of interest in this field since the meeting in 2001. The growing interest in the subject can also be seen when the most productive countries are analyzed. Data analysis of productivity parameters shows that research groups from Spain maintain a leadership position in research productivity at the level of European allergy meetings. The tendency of only a relatively small number of countries contributing the majority of research at the three congresses can also be remarkably illustrated by density-equalizing mapping procedures.

Whereas the number of published items was currently considered as an index of quantity of research productivity, the average citation per item may be used as an indicator for research quality. However, this approach is not available in the current study since the meeting abstracts are not listed in the PubMed online library and therefore not cited by many articles. However, online database-related studies have performed citation analyses for subfields of allergy research such as animal models of asthma [15]. In these studies it was shown that there is a major difference between research quantity as assessed by numbers of published items and research quality as assessed by citation parameters. Data was retrieved from the Thomson Institute for Scientific Information database Web of Science [18]. During the period from 1900 to 2006 a number of 3489 filed items were connected to animal models of asthma, the first being published in the year 1968 [15]. The studies were published by 52 countries with the US, Japan and the UK being the most productive suppliers, representing 55.8% of all published items. Analyzing the average citation per item as an indicator for research quality, Switzerland ranked first (30.54/item) and New Zealand ranked second for countries with more than 10 published studies [15]. With regard to the differences in this study on animal models, one can not draw any implications for the quality of science at the three currently analyzed allergy meetings. For this purpose, citation analyzing procedures are required that can not be applied in the present analysis of abstract books.

For the present study, it is important to realise that the analysis of European meetings are not representative for global allergy research. In this respect, a bias is represented by the host countries. Each of the three host countries is over-represented in its own meeting. This is a common phenomenon of conferences. Therefore, future studies using online data bases such as the PubMed or the Thomson Institute for Scientific Information database Web of Science [18] might be used to generate an overview of global allergy research activities using previously described techniques encompassing both scientometric and visualizing tools [19-22]. This might be performed within the NewQIS platform [23,24].

## Conclusion

The present study represents the first detailed bibliometric analysis of European allergy research. The data shows a strong increase in research productivity. In future, internationally established databases such as the Web of Science or the PubMed should be analyzed in combination with novel tools such as density- equalizing mapping.

## Competing interests

The authors declare that they have no competing interests.

## Authors' contributions

BGK, CS, and DQ designed the study. CS, MS, AS, and KA performed the search routines and constructed the different data files. DQ and KV performed pilot data search routines and analysis. CS, DQ, MS, AS, KA, TCF, TZ, KV and BGK participated in the discussion of the data and manuscript drafting. All authors have read and approved the final version of the manuscript.
